# Psychobiological Effects of Choral Singing on Affective State, Social Connectedness, and Stress: Influences of Singing Activity and Time Course

**DOI:** 10.3389/fnbeh.2018.00223

**Published:** 2018-09-27

**Authors:** Antje Bullack, Carolin Gass, Urs M. Nater, Gunter Kreutz

**Affiliations:** ^1^Department of Music, Carl von Ossietzky University Oldenburg, Oldenburg, Germany; ^2^Department of Psychology, University of Vienna, Vienna, Austria

**Keywords:** choral singing, positive and negative affect, social connectedness, cortisol, alpha-amylase, mental health, social support

## Abstract

Previous studies have suggested that there are complex psychobiological effects of amateur choral singing on well-being. Here, we investigate the influences of singing vs. non-singing on psychological and biological measurements, reflecting current positive and negative affect, perceived social connectedness, and physiological stress. We hypothesized that active singing leads to significant increases in these measurements compared to participating without singing. Amateur choristers (Exp. 1: *N* = 54, age range 18–85 years and Exp. 2: *N* = 49, age range 18–85 years) were tested in two experiments in which approximately half of the group was asked not to sing over periods of 30 (Exp. 1) and 60 min (Exp. 2), while the other half of the group sang. Dependent measures included scales for positive and negative affect and perceived social connectedness. In addition, saliva samples were collected to assess cortisol and alpha-amylase. The results revealed that singing activity had positive influences on affect measurements. However, significant increases in perceived social connectedness for singing were found only in Exp. 2. Biomarker changes were not significant across the experiments. Together, our findings suggest that both singing activity and duration of singing modulate psychological effects, with perceived social connectedness evolving over larger time spans than 30 min. Findings support the notion of beneficial psychological effects also for individuals, who report lower levels of general social support. The unexpected absence of biological effects warrants further investigation.

## Introduction

Recent surveys estimate that there are 37 million amateur choral singers in Europe, which means that 4.5% of the European population engage in active singing on a regular basis (European Choral Association, 2015). Clearly, such engagement raises questions about the motivations and reciprocal effects of choral singing on individual choristers. Seminal studies at the beginning of the millennium (Clift and Hancox, 2001; Bailey and Davidson, 2002) have initiated sustained research interest in amateur singing, in general (Müller and Lindenberger, 2011; Clift, 2012a,b), and its psychobiological effects on the singers, in particular (e.g., Beck et al., 2000; Grape et al., 2003; Kreutz et al., 2004; Kreutz, 2014; Fancourt et al., 2015, 2016).

One recurrent finding is that regular choral singing is associated with enhanced perceptions of positive affect, well-being, and quality of life, including among older adults (Clift and Hancox, 2010; Clift et al., 2010a; Livesey et al., 2012). In this vein, several studies have identified substantial health implications of regular engagement in singing (e.g., Skingley and Bungay, 2010; Skingley et al., 2011; Coulton et al., 2015). Systematic reviews of the literature ascribe potential benefits of group singing to a range of mental and physical health problems (Clift et al., 2010b; Clark and Harding, 2012).

Substantial progress in the field over the past decades has helped to identify some factors that might contribute to the positive—and in a few cases, the negative—effects of choir singing. For example, there is initial evidence that exposure to music is associated with different patterns of psychobiological changes compared to singing (Kreutz et al., 2004). Further studies have identified the importance of singing proficiency (Grape et al., 2003), size of the singing group (Weinstein et al., 2016), and particularly, a range of factors surrounding the *social* nature of the singing activity (Tarr et al., 2014; Pearce et al., 2015, 2016), which seem to significantly affect the reciprocal effects of the singing activity on the singers' organism. However, note that in all positive outcomes of choir singing, social problems in amateur choral societies may well compromise at least some of the potential benefits (Kreutz and Brünger, 2012a). Moreover, it cannot be overlooked that there is a strong gender bias favoring a 2:1 ratio of female vs. male participation in choral singing across Europe (see Kreutz and Brünger, 2012b for data from a German sample of over 3100 choristers) and that perceived general health and perceived singing related health has been correlated for female singers only (Clift et al., 2007). In sum, variables related to the social structure of choral societies might systematically influence singing as a communal experience.

Previous work points to the importance of individual differences, in terms of general, physical and mental health, as well to perceived social connectedness (SOC), as crucial variables that may also underlie the beneficial effects of choral singing. Nevertheless, many questions related to the factors and mechanisms that might modulate or even cause individual changes in psychophysiological measurements in response to singing have been only partially answered. For example, studies that are based on pre-post designs in single group singing sessions are inconsistent in the duration of the singing intervention. The duration of the interventions differed considerably across the studies, ranging from 20 min (Schladt et al., 2017) to 30 min (Valentine and Evans, 2001; Unwin et al., 2002; Grape et al., 2003), and up to 60 min and more (Beck et al., 2000; Kreutz et al., 2004; Kreutz, 2014; Sanal and Gorsev, 2014; Fancourt et al., 2015, 2016). While short-term changes in affect seem well-documented, the time effects of singing on social connectedness have been neglected (Tarr et al., 2014).

Another factor that has been neglected, to some extent, in the quasi-experimental approaches to choral singing is the singing activity itself. Previous studies have compared singing conditions, for example, with music listening (Kreutz et al., 2004), chatting (Kreutz, 2014), and no activity (Sanal and Gorsev, 2014). However, these studies leave open the question of whether the mere physical presence in a choir without singing produces similar effects as active singing. Therefore, it appears of interest to systematically investigate the associated psychological and social feelings in singing and non-singing choristers.

There is a range of evidence linking singing to physiological health benefits, such as strengthening the vocal apparatus, cardiorespiratory functions, and neurological networks related to endocrine stress and immune systems (for review, see Kang et al., 2017). For example, it appears to be well-established that cortisol (CORT) is a hormone associated with emotional stress and a valid measure for hypothalamus–pituitary–adrenal axis (HPA) activity (Kirschbaum and Hellhammer, 1994). Decreases of salivary cortisol have been found in low-stress singing conditions, while high-stress conditions (e.g., performances) are often connected with increased cortisol levels (Beck et al., 2000; Fancourt et al., 2015, 2016; Schladt et al., 2017). Furthermore, salivary alpha-amylase (sAA) is a psychobiological parameter that is known to reflect stress-related changes in the autonomic nervous system (ANS; Rohleder et al., 2004; Nater and Rohleder, 2009). For example Nater et al. (2005), found marked increases in sAA levels in participants exposed to the Trier Social Stress Test. Sanal and Gorsev (2014) presented one of the few studies that assessed changes in sAA during singing activity. There were different patterns of changes between the singing group and controls, however, these were attributable to group-differences in baseline measurements and not to the singing activity itself.

Here, we ask to what extent the perceived psychological and biological changes, as measured by concentrations of salivary cortisol and salivary alpha-amylase during group singing, are modulated by (a) the duration and time course of the activity and (b) the singing activity *per se* (i.e., the presence in the singing group while singing or not singing). Previous work has shown that listening to music in a choral rehearsal situation may interfere with the motivation for singing and, as a result, negatively influence the psychological affect (Kreutz et al., 2004); the social and biopsychological implications have remained unexplored.

### Aims and hypotheses

Based on these findings, the present study investigates the effects of singing vs. non-singing on psychological and biological measures over periods of 30 (Experiment 1) and 60 min (Experiment 2). To the best of our knowledge, this study is the first to compare singing and non-singing conditions in a naturalistic choir setting, with no between-group differences, other than the singing activity. We hypothesized that singing compared to non-singing would result in higher ratings of positive affect (PA), lower ratings of negative affect (NA), stronger perceived social connectedness (SOC), and lower levels of biological stress markers, namely, salivary cortisol (CORT) and salivary alpha-amylase (sAA). Furthermore, more pronounced results were expected after 60 min compared to after 30 min.

## Experiment 1

### Materials and methods

#### Participants

G^*^Power (Erdfelder et al., 1996) was used to conduct an a priori power analysis using the *F*-tests function and the algorithm for ANOVA (repeated measures, within-between interactions). According to this programme, a total sample size of 54 participants was needed to obtain an effect size of *f* = 0.25 (α-level: 0.05, power (1–β): 0.95, correlations among repeated measures: 0.5). Fifty-four adults (mean age = 59.63 years, *SD* = 15.01 years; range 18–85 years; 43 females) were recruited from an amateur choir that met weekly. They were randomized into a singing group (SIG; *n* = 31; 26 females) and a non-singing group (NSG; *n* = 23; 17 females), and the equal distribution of every voice type was considered. The SIG consisted of 12 sopranos, 12 altos, two tenors and five bass singers, while the NSG contained seven sopranos, ten altos, two tenors and four bass singers. Twenty-three participants reported taking pharmaceutical medication, 4 participants suffered from an acute illness, and 17 suffered from a chronic health condition, including Hashimoto's thyroiditis, hypothyreosis, fibromyalgia, lichen ruber, and asthma.

#### Measurement instruments

##### Questionnaires

A brief questionnaire with information about sociodemographic variables, music experience, and health condition was developed. Sociodemographic questions contained information about age, sex, family status, educational achievement, and profession. Music background was ascertained through questions about practical music lessons and choir experiences. In addition, information was gathered about acute and chronic diseases, as well as information about medication. Finally, the frequency of smoking and consumption of alcoholic drinks, sleep problems, as well as weight fluctuations, were ascertained.

Quality of life was assessed through the German version of the 12-item Short Form Health Survey (SF-12; Morfeld et al., 2011). The survey consists of 12 self-rated items measuring the physical component score (PCS) and the mental component score (MCS) of health-related quality-of-life over the last 4 weeks. The scores for both physical and mental health scales could range between 0 and 100, and a higher score indicates a better health status.

Perceived emotional and instrumental social support in everyday life was compiled using a short form of the Social Support Questionnaire (F-SozU, K-22; Fydrich et al., 2007). The participants were asked to evaluate (on a five-point Likert scale) how accurately each of the 22 items described the support they received. A rating of five points indicated a high perception of general social support.

The participants reported positive affect (PA) and negative affect (NA) through a total of six items, three representing PA (i.e., “I am feeling well,” “I am in good spirits,” “I am feeling unconcerned”) and three representing NA (i.e., “I am feeling tired,” “I am feeling bored,” “I am feeling stressed”). Each item was rated on a four-point Likert scale, ranging from “not at all” (0), “slightly” (1), “somewhat” (2), to “very strongly” (3). Therefore, higher scores indicated a stronger perception of either affect.

Perceived social connectedness (SOC) was measured through the Inclusion of Community in Self Scale (ICS; Mashek et al., 2007). The scale consists of one single item, showing six pairs of overlapping circles, with each pair overlapping slightly more than the previous pair. Whereas, one circle represents the participant, the other circle represents the community. The participant denotes the pair of circles that best represents their perception of connectedness to the people around them. The scores could range from 1 (= no perception of inclusion) to 6 (= high perception of inclusion).

##### Saliva collection and laboratory tests

As sampling devices, 0.5 ml Eppendorf LoBind® tubes were used. Saliva samples were collected following the guidelines of the biochemical laboratory of the University of Marburg (now at the University of Vienna). Therefore, the participants were instructed to swallow the entire saliva at the beginning. Afterwards, they accumulated saliva in their mouth cavity for a duration of 2 min. The participants then transferred the entire saliva into the tubes using a straw. The investigator attended the sampling process and supervised the procedure. As soon as possible after the rehearsal, the tubes were stored in a freezer and remained refrigerated until shipping to the laboratory. To determine the salivary flow, the tubes were weighed before and after the saliva collection. Cortisol levels (CORT) were measured using a commercially available enzyme-linked immunoassay (IBL, Hamburg, Germany). Salivary alpha-amylase (sAA) activity was measured using a kinetic colorimetric test and reagents obtained from Roche (Fa. Roche Diagnostics, Mannheim, Germany). Inter- and intraassay variance was below 10.00% for both CORT and sAA.

#### Procedures

The participants were tested during their regular weekly rehearsals. One week before the testing, they were informed about the test procedure and completed the sociodemographic questionnaire, as well as the Social Support Questionnaire (F-SozU). All participants provided informed consent individually. In addition, the saliva collection procedure was practiced to become accustomed to the process. Furthermore, the participants were instructed to avoid sportive activities, smoking, chewing gum and alcoholic drinks, as well as coffee, tea, juice, or soft drinks, during the day of the next rehearsal. In addition, they were asked to refrain from meals and tooth brushing at least 1 h prior to testing.

In the following week, Experiment 1 was conducted. Before the rehearsal started, each participant completed a short questionnaire regarding acute illness, sportive activities, ingested medication, and consumed drinks, as well as meals, during the day. Moreover, all choristers submitted their first saliva sample and completed the first questionnaire about their positive affect (PA) and negative affect (NA) and the perceived social connectedness (SOC). These measures served as the baseline (T1). Then, the group was randomly divided into singers (SIG) and non-singers (NSG), and the rehearsal started as usual. Both groups were asked to follow the instructions of the conductor (e.g., to sit up straight or to read the music), except that the NSG was told to not participate in the singing activity. The singing group conducted warm-up vocal exercises and rehearsed the oratorio “Messiah” (HWV 56) by George Frideric Handel. After 30 min, a second saliva sample was submitted, and the questionnaires on current affective states and perceived social connectedness were completed (T2).

This study was performed in accordance with the recommendations of the Carl von Ossietzky University's Ethics Committee. This committee approved the protocol of the current study. All subjects provided written informed consent, in accordance with the Declaration of Helsinki.

#### Statistical analysis

Physical and mental health component scores of the SF-12 were analyzed using the provided SPSS syntax file. The F-SozU scores were calculated as the mean of summed scores. To analyse the affect ratings, the values of the three items representing negative affect and positive affect were averaged for all respective time points.

The cortisol levels were converted from μg/dl into nmol/l. To consider the salivary flow rate, alpha-amylase was computed as U/min by multiplying the flow rate (ml/min) by the amylase concentrations (U/ml) of the sample. To achieve a normal distribution, the cortisol and amylase levels were log-transformed using the formula ln (x) + 10.

Pearson's bivariate correlation matrix was applied to assess possible significant correlations among variables used. All ratings and measures were analyzed using a 2 x 2 repeated measures analysis of variance (ANOVA) with Group (SIG/ NSG) as the between-subject factor and time (T1/T2) as the within-subject factor. Sex (female/male), intake of medication (no medication/ medication), and acute disease (no disease/ disease), as well as a chronic illness (no illness/illness), were entered as between-subject factors in a previous analysis to assess any influence from these variables. In case of SOC, an additional ANCOVA was conducted, using the same factors as described above but with the general perceived social support (F-SozU) as a covariate.

The preconditions for conducting ANOVAs were assessed (normality Box's M test of equality of covariance matrices and Mauchly's test of sphericity). Accordingly, degrees of freedom were estimated in the *F*-statistics using Greenhouse-Geisser corrections, as appropriate. Bonferroni's test was used for *post-hoc* comparisons of the means. The follow-up analysis was performed using independent samples *t*-tests and paired samples *t*-tests. As directed hypotheses were formulated, one-tailed significance levels were employed. In all statistical tests, the *p*-values were set to 0.05. In addition, partial eta-squared was calculated as a measure of the effect size.

Due to incomplete data, one participant had to be excluded from further analysis regarding negative affect, and three participants were excluded from the analysis of the perceived social connectedness. Furthermore, because of unusual results concerning the intake of food within the last hour before testing, three participants were excluded from the analysis of cortisol measures, as well as eight participants from the analysis of alpha-amylase.

### Results

Table 1 shows the means and standard deviations for psychological and physiological measures during time points separate for singers and non-singers. No between-group differences regarding physical or mental health scores, general social support, positive and negative affect, perceived social connectedness or physiological measures were found at T1, all *t*s ≤ 1.98, all *p*s ≥ 0.10). Nevertheless, a non-significant trend indicated a difference of the groups regarding sAA levels at T1, *t*_(45)_ = 1.98, *p* = 0.054, *d* = 0.59. The sAA values of the singing group were higher at baseline compared to the non-singers, and the difference indicated a medium size effect.

**Table 1:** The means (and standard deviations) of the psychological and physiological measurements in Experiment 1 for the singing group (SIG) and non-singing group (NSG) at baseline (T1) and after 30 min (T2).

	**T1**	**T2**
	**M (SD)**	**M (SD)**
**PA**		
SIG	1.91 (0.75)	2.26 (0.74)
NSG	1.97 (0.63)	2.03 (0.61)
Total	1.94 (0.70)	2.16 (0.70)
**NA**		
SIG	0.95 (0.63)	0.54 (0.56)
NSG	1.02 (0.50)	1.00 (0.53)
Total	0.97 (0.57)	0.73 (0.59)
**SOC**		
SIG	3.61 (1.42)	4.93 (1.31)
NSG	3.78 (1.13)	4.04 (1.15)
Total	3.69 (1.29)	4.24 (1.24)
**CORT**		
SIG	11.67 (0.63)	11.56 (0.67)
NSG	11.48 (0.56)	11.52 (0.43)
Total	11.59 (0.60)	11.55 (0.57)
**sAA**		
SIG	13.46 (0.87)	13.23 (1.21)
NSG	12.95 (0.97)	12.67 (0.91)
Total	13.26 (0.93)	13.01 (1.13)

*SIG, singing group; NSG, non-singing group; PA, positive affect; NA, negative affect; SOC, social connectedness; CORT, salivary cortisol; sAA, salivary alpha-amylase*.

Neither sex, intake of medication nor chronic and acute diseases yielded any interaction effects on the dependent measures in this study and, thus, were not considered in further analyses, all *F*s ≤ 3.22, all *p*s ≥ 0.08.

#### Health status and social support

The self-reported physical health scores (PCS) ranged from 23 to 60 (*M* = 48.96, *SD* = 9.15), and the respective mental health scores (MCS) ranged from 26 to 62 (*M* = 49.12, *SD* = 8.82). As one would expect, a negative correlation between age and PCS was found, *r*_(51)_ = −0.30, *p* < 0.05. Scores of the Social Support Questionnaire (F-SozU) ranged between 1.64 and 4.95 (*M* = 4.11, *SD* = 0.71), indicating a high general social support score. Furthermore, a positive correlation between the general social support and the PCS, *r*_(51)_ = 0.33, *p* < 0.05, as well as the MCS, *r*_(51)_ = 0.49, *p* < 0.001, was found.

#### Psychological measures

**Figure 1:**
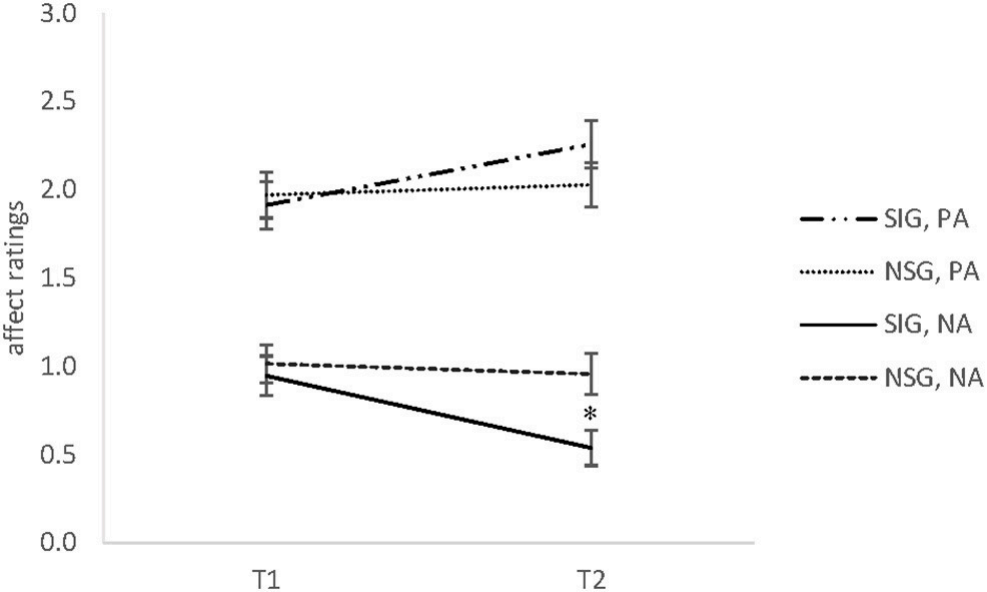
The means and standard error of the mean (SEM) of positive affect (PA) and negative affect (NA) ratings in Experiment 1 for the singing group (SIG) and non-singing group (NSG) at baseline (T1) and after 30 min (T2). SIG, singing group; NSG, non-singing group; PA, positive affect; NA, negative affect (range 0–3). *p < 0.05.

A time x group interaction for positive affect indicated a non-significant trend, *F*_(1, 52)_ = 3.77, *p* = 0.06, $\eta_{p}^{2}$ = 0.07, however, pairwise comparisons revealed no significant differences between groups, all *t*s ≤ 1.21, all *p*s ≥ 0.23. However, there was a significant main effect of time, *F*_(1, 52)_ = 7.44, *p* < 0.01, $\eta_{p}^{2}$ = 0.13. The Bonferroni-adjusted *post-hoc* analysis revealed a significant increase between the T1 [95% CI (1.75, 2.14)] and T2 [95% CI (1.95, 2.33)]. Even so, as depicted in Figure 1, in-group comparisons showed a significant increase of PA in the singing group only, *t*_(30)_ = 4.17, *p* < 0.001, *d* = 0.75, whereas the non-singing group showed no such changes, *t*_(30)_ = 0.45, *p* = 0.66.

A significant time x group interaction was observed for negative affect, *F*_(1, 51)_ = 5.24, *p* < 0.05, $\eta_{p}^{2}$ = 0.09. Comparisons of the means revealed no significant between-group differences at baseline, whereas the singing group showed significantly lower values at T2 compared to the non-singing group, *t*_(52)_ = 2.73, *p* < 0.01, *d* = 0.75. In addition, a significant decrease of NA over time was found for the singers, *t*_(30)_ = 4.00, *p* < 0.001, *d* = 0.71. In contrast, the non-singers indicated no such changes of negative affect, *t*_(21)_ = 0.11, *p* = 0.92 (cf. Figure 1).

The ANOVA for perceived social connectedness (SOC) produced no significant interactions, however, there was a significant main effect of time, *F*_(1, 49)_ = 13.79, *p* ≤ 0.001, $\eta_{p}^{2}$ = 0.22. The Bonferroni-adjusted *post-hoc* analysis revealed higher ratings at T2 [95% CI (3.88, 4.57)] compared to T1 [95% CI (3.33, 4.06)]. There was a non-significant trend indicating a time x group interaction effect, *F*_(1, 49)_ = 3.47, *p* = 0.07, $\eta_{p}^{2}=$ 0.07. Comparison of the means between groups revealed no significant differences, all *t*s ≤ 0.99, all *p*s ≥ 0.32. However, in-group comparisons revealed a significant increase of SOC values in singing group, *t*_(27)_ = 4.35, *p* < 0.001, *d* = 0.82, whereas SOC remained level for the non-singers, *t*_(22)_ = 1.19, *p* = 0.25.

Finally, we analyzed whether the individual trait measures of social support (F-SozU) was systematically associated with the state changes of social connectedness. Therefore, we included the F-SozU measure as a covariate in an additional ANOVA. However, the inclusion of this independent measure did not alter these results, *F*_(1, 48)_ = 0.77, *p* = 0.39, $\eta_{p}^{2}$ = 0.02.

#### Physiological measurements

The ANOVAs for cortisol (CORT) or salivary alpha-amylase (sAA) revealed no significant time x group interactions, all *F*s ≤ 4.00, all *p*s ≥ 0.10. The only exception was a non-significant trend for a main effect of time for sAA, *F*_(1, 42)_ = 4.00, *p* = 0.052, $\eta_{p}^{2}$ = 0.08, indicating a decrease of sAA across groups. Nevertheless, the Bonferroni-adjusted *post-hoc* analysis revealed no significant differences between T1 and T2.

### Discussion

We asked how singing activity compared to non-singing modulates psychological and biological effects in amateur choristers over a 30-min period. First, the hypothesis that the singing group showed significantly improved affective states compared to the non-singing group after the rehearsal was partially confirmed. There was a non-significant trend in positive affect (PA), as well as a significant difference in negative affect (NA) across groups, indicating that singing activity can positively impact well-being by reducing negative emotions. This outcome is further supported by the observation that changes in both dimensions of affect were found in active singers only. The findings align with previous research by Sanal and Gorsev (2014), who also found changes only in the negative affect. These authors emphasized the idea of differential assessments of positive and negative affect. In addition, our results show that changes were caused by singing activity only, while no changes were found through mere exposure.

Second, the results indicate no between-group differences regarding the perceived social connectedness (SOC) but instead a general increase over time. There was a non-significant trend indicating an increase of SOC values in the singing group but not in the non-singing group. Therefore, the members of the latter group did not develop strong feelings of inclusion or exclusion, which suggests that lack of singing activity was at least not detrimental to group participation, *per se*. However, singing might have strengthened individual feelings of belonging to that group, although the short duration of the intervention may have prevented reported values to reach significance levels. In sum, these findings do not provide strong support for the hypothesis that singing would lead to significantly enhanced perceptions of social connectedness.

Finally, and contrary to expectations, no changes in the stress markers salivary cortisol (CORT) and salivary alpha-amylase (sAA) were found across time and groups. Although the sAA levels decreased during the choir rehearsal, this trend was not significant. It is possible that the duration of the intervention had to be extended to observe any significant changes in these measures.

Together, there is some evidence to suggest that singing activity particularly influences psychological measures. However, the results regarding PA, as well as perceived SOC, were not as distinct as expected, compared to previous findings (Valentine and Evans, 2001; Unwin et al., 2002; Grape et al., 2003). Likewise, the sAA levels seemed to change during the rehearsal, even though independent of the group. The occurring trends could be an indicator that the narrow timeframe of 30 min was insufficient to produce any significant effects. Therefore, Experiment 2 was designed to replicate the findings of Experiment 1 and to extend the intervention duration to 60 min.

## Experiment 2

### Materials and methods

#### Participants

A priori power analysis revealed a necessary sample size of 44 participants to achieve a medium effect size (*f* = 0.25), with a repeated measures ANOVA and a within-between subject design [α-level: 0.05, power (1–β): 0.95, correlations among repeated measures: 0.5]. Forty-nine adults (mean age = 57.69 years, *SD* = 14.89 years, range 18–85 years, 39 females) were recruited from the same choir as that in Experiment 1. Within this cohort, 45 participants (35 females) were identical in both sessions. They were randomized into a singing group (SIG; *n* = 31; 25 females) and a non-singing group (NSG; *n* = 18; 14 females), and the equal distribution of every voice type was considered. However, due to the small quantity of tenor singers, all participants of this voice type were allocated to the SIG. The SIG consisted of 13 sopranos, 11 altos, three tenors and four bass singers, whereas the NSG contained five sopranos, nine altos, and four bass singers. Twenty-one participants reported to be taking pharmaceutical medications, 3 participants suffered from an acute illness, and 16 suffered from a chronic health condition, including Hashimoto's thyroiditis, hypothyreosis, Lichen ruber, asthma, and cancer.

#### Measurement instruments

The same measurement instruments used in Experiment 1 were used in Experiment 2.

#### Procedure

Experiment 2 was conducted 1 week after Experiment 1. The same procedure as that used in Experiment 1 was followed, except that an additional time point after 60 min was included (T3). Again, the session involved the rehearsal of Handel's “Messiah” (see section Procedures, for details).

#### Statistical analysis

Data preparation was conducted in the same manner as in Experiment 1. Pearson's bivariate correlation matrix was applied to assess any possible significant correlations among variables used. All ratings and measures were analyzed through 2 x 3 repeated measures ANOVAs with group (SIG/ NSG) as the between-subject factor and time (T1/T2/T3) as the within-subject factor. Sex (female/ male), intake of medication (no medication/ medication) and acute disease (no disease/ disease), as well as a chronic health condition (no illness/ illness), were entered as the between-subject factors in a previous analysis to assess any influence of these independent variables. Again, an additional ANCOVA was conducted for perceived social connectedness, with social support (F-SozU) as a covariate.

The preconditions for conducting ANOVAs were assessed (normality Box's M test of equality of covariance matrices and Mauchly's test of sphericity). Accordingly, degrees of freedom were estimated in the *F*-statistics using Greenhouse-Geisser corrections, as appropriate. Bonferroni's test was used for *post-hoc* comparisons of the means. In all statistical tests, *p*-values were set to 0.05. In addition, partial eta-squared was calculated as a measure of the effect size. Follow-up analysis was performed through independent samples *t*-tests and paired samples *t*-tests. As directed hypotheses were formulated, one-tailed significance levels were employed. In this case, *p*-values were adjusted to 0.033 (a significance level of 0.1 divided by 3 corresponding to the three tests conducted).

Due to incomplete data, one participant had to be excluded from further analysis regarding negative affect and positive affect, and three participants were excluded from analysis of the perceived social connectedness. Furthermore, four participants were excluded from the analysis of salivary cortisol measures, as well as five participants from analysis of salivary alpha-amylase, because of unusual values.

### Results

Table 2 shows the means and standard deviations for psychological and physiological measures during time points separate for singers (SIG) and non-singers (NSG). No between-group differences regarding physical or mental health component scores, general social support, positive and negative affect, perceived social connectedness or physiological measures were found, all *t*s ≤ 1.47, all *p*s ≥ 0.15. Sex, medication, chronic or acute diseases exerted non-significant influences, all *F*s ≤ 1.27, all *p*s ≥ 0.29. Therefore, these variables were not considered further.

**Table 2:** The means (and standard deviations) of the psychological and physiological measurements in Experiment 2 for the singing group (SIG) and non-singing group (NSG) at baseline (T1), after 30 min (T2), and after 60 min (T3).

	**T1**	**T2**	**T3**
	**M (SD)**	**M (SD)**	**M (SD)**
**PA**			
SIG	1.95 (0.64)	2.14 (0.61)	2.22 (0.59)
NSG	2.24 (0.51)	1.84 (0.69)	1.76 (0.63)
Total	2.05 (0.61)	2.03 (0.69)	2.06 (0.64)
**NA**			
SIG	0.78 (0.72)	0.49 (0.51)	0.67 (0.58)
NSG	0.86 (0.47)	1.22 (0.64)	1.63 (0.80)
Total	0.81 (0.64)	0.75 (0.65)	1.01 (0.80)
**SOC**			
SIG	3.24 (1.30)	4.00 (0.93)	4.41 (0.98)
NSG	3.81 (1.33)	3.50 (1.46)	3.50 (1.37)
Total	3.44 (1.329	3.82 (1.15)	4.09 (1.20)
**CORT**			
SIG	11.51 (0.55)	11.33 (0.65)	11.40 (0.62)
NSG	11.58 (0.39)	11.47 (0.55)	11.57 (0.55)
Total	11.54 (0.50)	11.38 (0.62)	11.46 (0.59)
**sAA**			
SIG	13.52 (0.83)	13.24 (0.96)	13.22 (0.94)
NSG	13.24 (1.32)	13.13 (1.40)	12.93 (1.08)
Total	13.42 (1.02)	13.20 (1.11)	13.12 (1.00)

*SIG, singing group; NSG, non-singing group; PA, positive affect; NA, negative affect; SOC, social connectedness; CORT, salivary cortisol; sAA, salivary alpha-amylase*.

#### Health status and social support

The self-reported physical health component scores (PCS) ranged from 23 to 60 (*M* = 50.06, *SD* = 8.72) and the mental health component scores (MCS) ranged from 23 to 62 (*M* = 48.95, *SD* = 9.20). Again, a negative correlation was observed between age and PCS, *r*_(46)_ = −0.30, *p* < 0.05. Scores from the Social Support Questionnaire (F-SozU) ranged from 1.64 to 4.95 (*M* = 4.18, *SD* = 0.65). Furthermore, a positive correlation was observed between the general social support and the MCS, *r*_(46)_ = 0.53, *p* < 0.001.

#### Psychological measures

**Figure 2:**
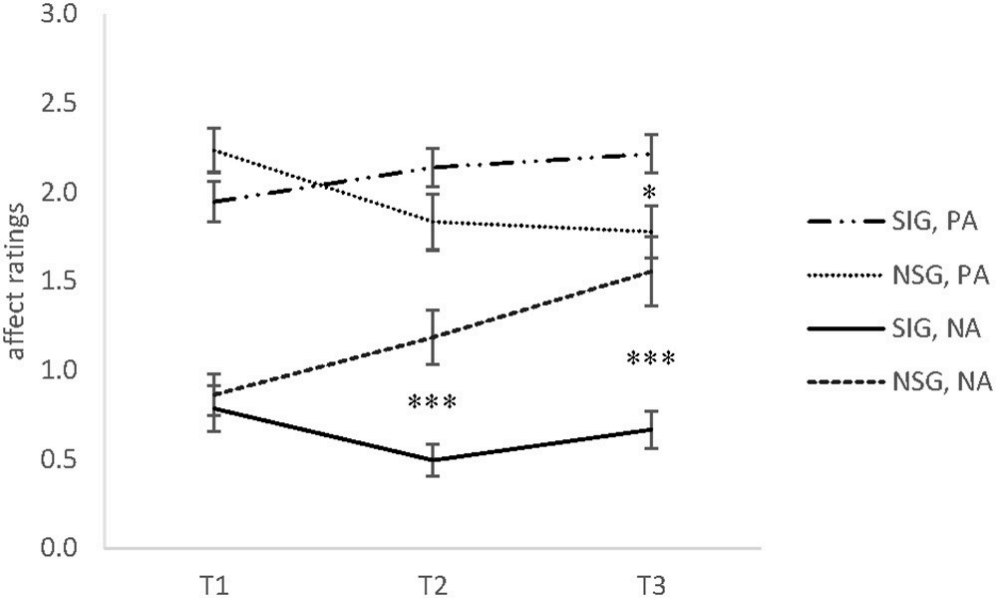
The means and standard error of the mean (SEM) of positive affect (PA) and negative affect (NA) Ratings in Experiment 2 for the singing group (SIG) and non-singing group (NSG) at baseline (T1), after 30 min (T2), and after 60 min (T3). SIG, singing group; NSG, non-singing group; PA, positive affect; NA, negative affect (range 0–3). *p < 0.05, ***p < 0.001.

There was a significant time x group interaction effect for positive affect (PA), *F*_(1.51, 69.25)_ = 12.16, *p* < 0.001, $\eta_{p}^{2}$ = 0.21. Comparisons of the means were significant between groups at T3, *t*_(47)_ = 2.45, *p* < 0.033, *d* = 0.73. In-group comparisons revealed a non-significant increase between T1 and T3 for the singing group, *t*_(30)_ = 2.17, *p* = 0.04, *d* = 0.44, whereas the non-singing group showed a decrease of PA between T1 and T2, *t*_(16)_ = 2.91, *p* < 0.033, *d* = 0.71, and from T1 to T3, *t*_(16)_ = 3.77, *p* < 0.006, *d* = 0.92 (see Figure 2).

A significant time x group interaction was also found for negative affect (NA), *F*_(1.62, 74.70)_ = 10.77, *p* < 0.001, $\eta_{p}^{2}$ = 0.19. Comparison of the means revealed significant differences between groups at T2, *t*_(47)_ = 4.17, *p* < 0.0006, *d* = 1.24, and T3, *t*_(47)_ = 4.41, *p* < 0.0006, *d* = 1.31. The singers showed a significant decrease of perceived NA between T1 and T2, *t*_(30)_ = 2.87, *p* < 0.033, *d* = 0.68. Figure 2 shows that NA in the non-singing group increased between T2 and T3, *t*_(17)_ = 2.87, *p* < 0.033, *d* = 0.52, and from T1 to T3, *t*_(16)_ = 3.79, *p* < 0.006, *d* = 0.92.

**Figure 3:**
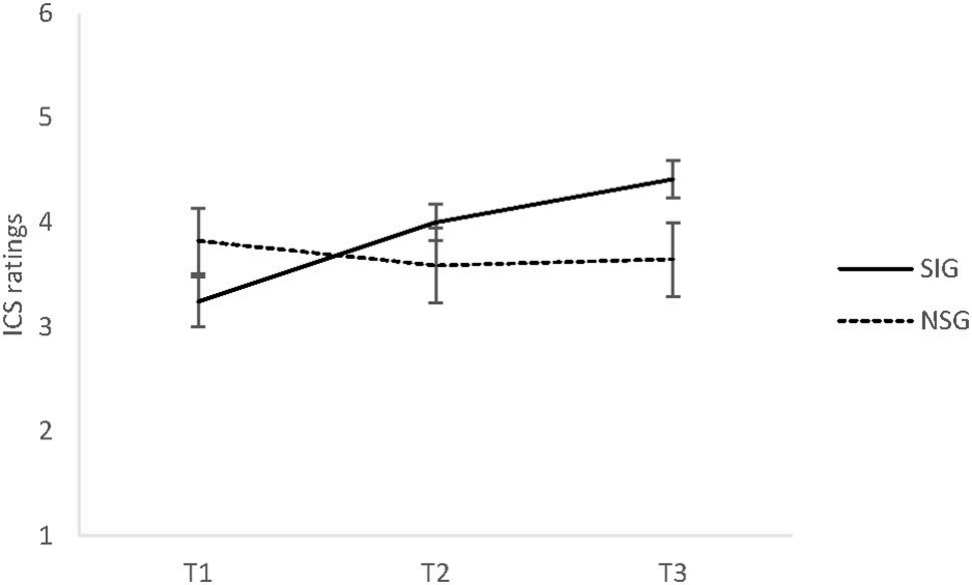
The means and standard error of the mean (SEM) of perceived social connectedness (SOC) in Experiment 2 for the singing group (SIG) and non-singing group (NSG) at baseline (T1), after 30 min (T2), and after 60 min (T3). SIG, singing group; NSG, non-singing group; ICS, inclusion of communityin self scale (range 1–6).

There was a significant time x group interaction for perceived social connectedness (SOC), *F*_(1.52, 65.53)_ = 10.60, *p* < 0.001, $\eta_{p}^{2}$ = 0.20. Pairwise comparisons revealed no significant between-group differences at all three time points, all *t*s ≤ 1.93, all *p*s ≥ 0.07. There was a non-significant trend indicating group differences at T3, *t*_(24.69)_ = 1.93, *p* = 0.07, *d* = 0.65 (see Figure 3) with higher ratings of SOC in the singing group. In addition, in-group comparisons revealed that the SIG showed significant increases between T1 and T2, *t*_(28)_ = 3.64, *p* < 0.006, *d* = 0.68, between T2 and T3, *t*_(28)_ = 3.04, *p* < 0.006, *d* = 0.56, and from T1 to T3, *t*_(28)_ = 5.03, *p* < 0.0006, *d* = 0.93. In contrast, no changes in the NSG occurred, all *t*s ≤ 0.96, all *p*s ≥ 0.35. Finally, no significant main effects of time or group on SOC were found, regardless of general social support, *F*_(1, 42)_ = 0.07, *p* = 0.80, $\eta_{p}^{2}$ = 0.002. The observed interaction was unaffected by the inclusion of the social support trait measure (F-SozU) as an independent variable.

#### Physiological measures

No interaction or main effects for the CORT measures were found, all *F*s ≤ 2.68, all *ps* ≥ 0.10. The only exception was a non-significant trend for the main effect of sAA, *F*_(2, 82)_ = 2.68, *p* = 0.07, $\eta_{p}^{2}$ = 0.06, indicating a decrease of sAA over time. Nevertheless, the Bonferroni-adjusted *post-hoc* analysis revealed no significant differences between time points.

### Discussion

We assumed that singing activity in comparison to non-singing led to better affect, stronger perceived social connectedness (SOC) and lower levels of biological stress markers during a 60 min rehearsal. Subsequent to Experiment 1, a replication of the previous findings, as well as more pronounced results after 60 min, were expected.

The results support the assumption of active singing as an effective strategy to reduce negative affect (NA). Surprisingly, and contrary to Experiment 1, active singing did not affect positive affect (PA), whereas the participants in the non-singing group experienced a decrease in positive effect. In addition, NA increased in this group and decreased in the singing group. It cannot be ruled out that the knowledge about the procedure carried over from the first experiment a week earlier, particularly in the NSG. The disappointment of being present during the choir rehearsal without being allowed to sing could have had an impact. Nonetheless, positive affect was stable in the singing group, which also benefited from decreases of negative affect. These findings corroborate those of previous research (Kreutz et al., 2004; Fancourt et al., 2016).

Furthermore, there was a non-significant trend indicating differences in perceived social connectedness between groups, with an increase in the singing group, whereas no such increase was found in the non-singing group. This could be an indication that joint singing benefits social bonding, although the results were not as strong as expected. To some extent, this result could be attributed to the specific choir group. Although the members of the participating choir fluctuate to a certain extent, most members have been involved in this group for a long time. Therefore, the SOC may not vary as much as it does in a newly formed singing group (Kreutz, 2014; Pearce et al., 2015).

Note that general social support appears to not compromise the evolving feeling of social connectedness. Previous studies have pointed out the psychological benefits of choral singing to individuals suffering from mental health problems (e.g., Coulton et al., 2015). Therefore, the degree to which the enhanced feelings can be sustained beyond over a prolonged period of time, for example, should be of interest in future studies.

Contrary to expectations, there were no changes in salivary cortisol (CORT) and salivary alpha-amylase (sAA) levels after 1 h of rehearsal. In sum, the results suggest that singing activity influences psychological measures but not physiological measures.

## General discussion

We investigated the effects of singing vs. non-singing on psychological and biological measures over periods of 30 (Experiment 1) and 60 (Experiment 2) min. We hypothesized that singing activity led in comparison to non-singing to better affect ratings, stronger perceived social connectedness (SOC) and a reduction of biological stress markers in salivary. In addition, more pronounced results were expected after a longer duration of singing respectively non-singing.

As expected, the participants in the singing group (SIG) showed better affect compared to participants in the non-singing group (NSG), and this difference was particularly marked by a decrease of negative affect (NA), which emphasizes the approach of considering positive and negative affect as two independent dimensions (Cohen and Pressman, 2006). These positive changes in affect caused by choir singing have been reported in previous studies (Grape et al., 2003; Clift et al., 2010a) and could be one important factor that connects choir singing with a positive impact on general psychological (Valentine and Evans, 2001; Clift and Hancox, 2010) and physiological health (Kreutz et al., 2004; Fancourt et al., 2016; Schladt et al., 2017).

In contrast to our findings, there were significant between-group differences only after 60 min when considering the perceived social connectedness. It can be assumed that familiarity between the choir members influenced the perceived SOC, which makes it harder to provoke changes in this measure. However, changes are attributable to the SIG, thus, the singing activity itself seems to have an influence on the perceived SOC. A new finding is that situational SOC during choir singing does not seem to be connected to the general perception of social support in everyday life, which could be evidence that a choir offers a special community that might have beneficial effects, particularly for people suffering from the negative effects of social exclusion (Dingle et al., 2012; Croom, 2015).

Furthermore, there has been a connection between general social support (F-SozU) and the mental health score (MCS). This relationship has been observed in previous studies and across different age groups (Everard et al., 2000; Bovier et al., 2004; Hawton et al., 2011). Thoits (2011) assumed that social relationships support mental health through stress-moderating effects. However, the causality and underlying mechanisms are still unclear. Specifically, the influence of singing activity to modulate not only temporary perceived social connectedness but also more general perceptions of general social support could be verified only by longitudinal studies that include a greater number of measurements and include data from every-day life situations.

Contrary to expectations, no changes in salivary cortisol levels (CORT) could be observed during the experiments. The stable level may be explained by a diurnal effect. While the CORT levels showed the highest concentration in the morning, they decreased during the waking hours and reached the lowest level in the evening (Schmidt-Reinwald et al., 1999). Given that the choir rehearsal took place between 8.30 and 9.30 p.m., it is possible that the secretion dynamics of CORT were restricted at this time of day. Furthermore, no changes were observed in salivary alpha-amylase (sAA). These results support the findings of Sanal and Gorsev (2014) and could lead to the assumption that the short-time interventions did not last long enough to influence the ANS (Nater et al., 2007). In addition, the study was conducted during a stressful rehearsal period, which could have had a general influence on biological stress markers. Nonetheless, the change of sAA during choir singing needs further study.

In sum, the two studies show that the singing activity of amateur choristers influences affect and perceived SOC. These measures could have an essential influence on mental health and well-being.

## Limitations

In this study, the participants were from a previously existing choir. Therefore, the study sample showed a strong heterogeneity with respect to age, education level, and health status. Furthermore, there was no balanced proportion of female and male participants. Nevertheless, the sample represents a naturalistic choir setting and reflects the pluralism of a choral group. In addition, we did not specify the singing repertoire, which was decided by the choral conductor, thus, the least possible influence on a regular choir rehearsal was provoked by the study situation. Nevertheless, the nature of the sung materials could have influenced our dependent measures.

The most critical point of this study was perhaps the demand characteristic, which required a group of choir members to refrain from singing over extended periods of time. We cannot ascertain to what extent each participant adhered to the task demands. Whereas, the members of the singing group did not sing continuously throughout the rehearsal sessions, which would have been unusual and unrealistic, the level of overt or covert engagement in singing among the non-singers was not controlled. Moreover, the task demands, *per se*, could have provoked negative feelings in individual participants, regardless of the familiar situation and shared environment. Therefore, measures of perceived stress would have been useful to complement the set of dependent variables of the present study. Finally, this study was designed to evaluate the short-term effects of choral singing, thus, it remained unclear whether long-term observations could lead to more pronounced changes.

## Conclusion

The results suggest that the singing activity of amateur choristers has differential effects on positive and negative affect, as well as perceived social connectedness, over time. The psychological effects were similar in participants with varying levels of self-reported mental health and general social support. However, no marked changes in salivary cortisol and alpha-amylase were found. The hypothesis of the positive biopsychological effects of singing is partially supported. These findings must be further explored and confirmed in future experiments.

## Author contributions

AB and GK conceived and designed the research; AB and CG collected the data; UN analyzed the salivary samples; AB and GK analyzed the data; and AB, GK, and UN wrote the manuscript.

### Conflict of interest statement

The authors declare that the research was conducted in the absence of any commercial or financial relationships that could be construed as a potential conflict of interest.
